# Supplementary material for: Interaction of tankyrase and peroxiredoxin II is indispensable for the survival of colorectal cancer cells
Source: Nat Commun. 2017 Jun 28;8:40. doi: 10.1038/s41467-017-00054-0 (PMC5489516; doi:10.1038/s41467-017-00054-0)

File name: Supplementary Information
Description: Supplementary figures.

File name: Peer review file
Description:

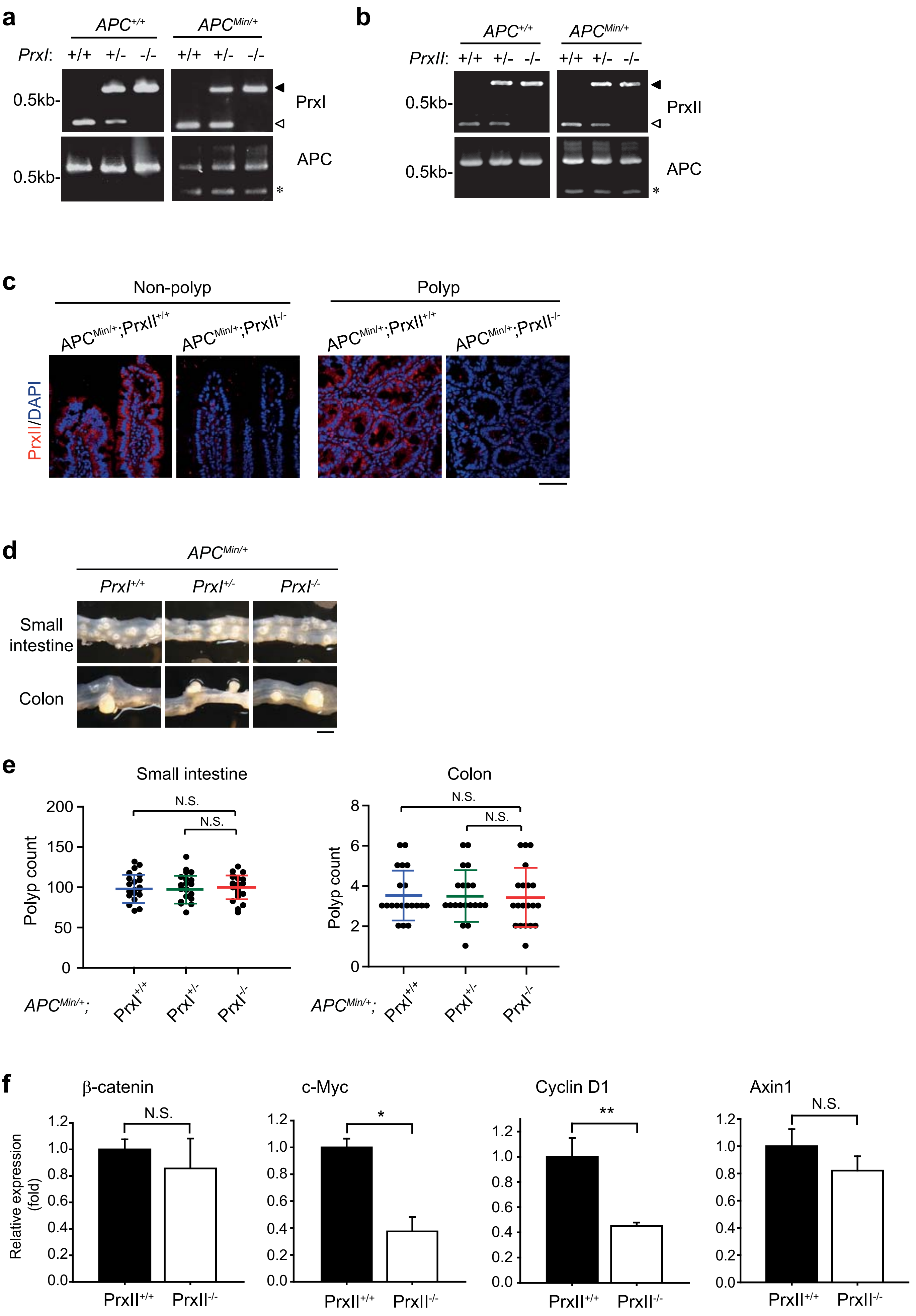
a
APC+/+
APCMin/+
PrxI: +/+ +/- -/- +/+ +/- -/-
0.5kb-
PrxI
0.5kb-
APC
*
b
APC+/+
APCMin/+
PrxII: +/+ +/- -/- +/+ +/- -/-
0.5kb-
PrxII
0.5kb-
APC
*
c
Non-polyp
Polyp
APCMin/+;PrxII+/+
APCMin/+;PrxII-/-
APCMin/+;PrxII+/+
APCMin/+;PrxII-/-
PrxII/DAPI
d
APCMin/+
PrxI+/+
PrxI+/-
PrxI-/-
Small intestine
Colon
e
Small intestine
Colon
Polyp count
200
150
100
50
0
8
6
4
2
0
N.S.
N.S.
APCMin/+;
PrxI+/+
PrxI+/-
PrxI-/-
f
β-catenin
c-Myc
Cyclin D1
Axin1
Relative expression (fold)
1.2
1.0
0.8
0.6
0.4
0.2
0.0
N.S.
*
**
PrxII+/+
PrxII-/-

Supplementary Fig. 1. Deletion of PrxI and PrxII in $APC^{Min/+}$ mice.

a and **b**. The double mutant mice were generated by mating $PrxI^{+/-}$ or $PrxII^{+/-}$ mice with $APC^{Min/+}$ mice. Mice were genotyped at 4 weeks by genomic PCR. The gene products for $PrxI/II^{+/+}$ and $PrxI/II^{-/-}$ mice are indicated by arrowheads (wild type, *closed arrowhead*; neo cassette, *filled arrowhead*). The gene product for truncated *APC* gene is indicated by an asterisk. The DNA size markers are labeled in kilobases (kb).

c. PrxII protein expression shown by immunofluorescence staining in non-polyp segments and polyps in the small intestine. Nuclei were stained with DAPI. Scale bar, 50 μm.

d. Representative photomicrographs of longitudinally-opened intestinal tissues from $APC^{Min/+};PrxI^{+/+}$, $APC^{Min/+};PrxI^{+/-}$, and $APC^{Min/+};PrxI^{-/-}$ mice. Scale bar, 5 mm.

e. The macroscopic polyps (> 3 mm in diameter) were counted in whole-mount intestinal tissues from $APC^{Min/+};PrxI^{+/+}$, $APC^{Min/+};PrxI^{+/-}$, and $APC^{Min/+};PrxI^{-/-}$ mice. Dot plots represent the average polyp numbers ± SD (n = 20 mice per group, P = 0.9144 (small intestine) and 0.7282 (colon) for $APC^{Min/+};PrxI^{+/+}$ vs $APC^{Min/+};PrxI^{-/-}$, P = 0.8592 (small intestine) and 0.8193 (colon) for $APC^{Min/+};PrxI^{+/-}$ vs $APC^{Min/+};PrxI^{-/-}$ with repeated measures ANOVA). N.S., not significant.

f. Expression of Axin1, β-catenin, and β-catenin target genes in polyps from $APC^{Min/+};PrxII^{+/+}$ and $APC^{Min/+};PrxII^{-/-}$ mice. Level of mRNA was measured by quantitative real-time PCR as described in *Methods*. Data in the graph are means ± SD of fold change in mRNA level versus that of $APC^{Min/+};PrxII^{+/+}$ mice (n = 4 mice per group; $^{*}P < 0.01$; $^{**}P < 0.001$). N.S., not significant.

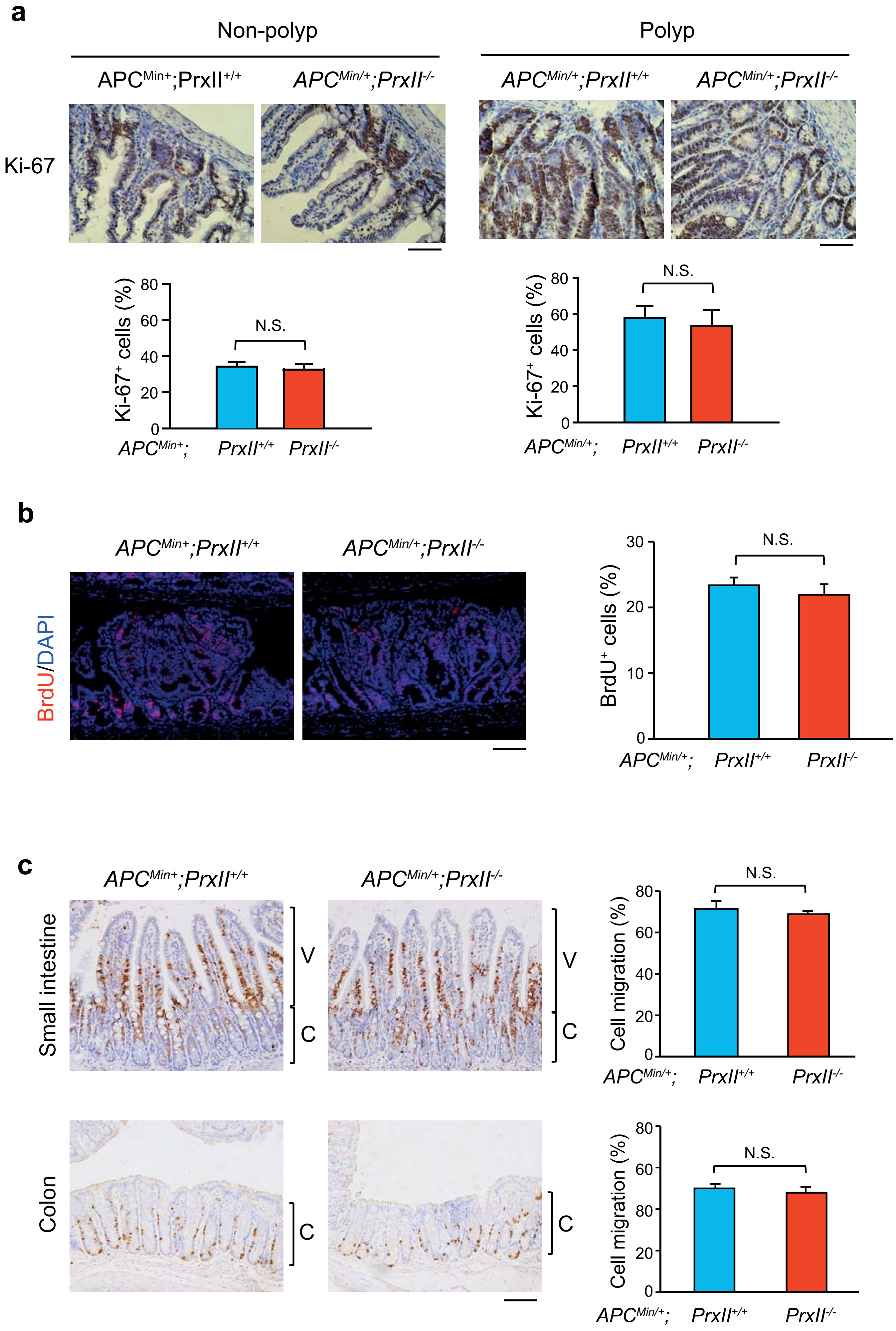
a
Non-polyp
Polyp
APC^{Min+};PrxII$^{+/+}$
APC$^{Min/+}$;PrxII$^{-/-}$
APC$^{Min/+}$;PrxII$^{+/+}$
APC$^{Min/+}$;PrxII$^{-/-}$
Ki-67
Ki-67^{+} cells (%)
0
20
40
60
80
N.S.
APC^{Min+};
PrxII$^{+/+}$
PrxII$^{-/-}$
Ki-67^{+} cells (%)
0
20
40
60
80
N.S.
APC$^{Min/+}$;
PrxII$^{+/+}$
PrxII$^{-/-}$
b
APC^{Min+};PrxII$^{+/+}$
APC$^{Min/+}$;PrxII$^{-/-}$
BrdU/DAPI
BrdU^{+} cells (%)
0
10
20
30
N.S.
APC$^{Min/+}$;
PrxII$^{+/+}$
PrxII$^{-/-}$
c
APC^{Min+};PrxII$^{+/+}$
APC$^{Min/+}$;PrxII$^{-/-}$
Small intestine
V
C
V
C
Cell migration (%)
0
20
40
60
80
N.S.
APC$^{Min/+}$;
PrxII$^{+/+}$
PrxII$^{-/-}$
Colon
C
C
Cell migration (%)
0
20
80
60
80
N.S.
APC$^{Min/+}$;
PrxII$^{+/+}$
PrxII$^{-/-}$

Supplementary Fig. 2. Proliferation and migration of epithelial cells in the intestinal polyps and villi of *APC*$^{Min/+}$;*PrxII*$^{+/+}$ and *APC*$^{Min/+}$;*PrxII*$^{-/-}$ mice.

a. Immunohistochemistry images for Ki-67 in the non-polyp segments and the polyps of *APC*$^{Min/+}$;*PrxII*$^{+/+}$ and *APC*$^{Min/+}$;*PrxII*$^{-/-}$ mice. DAB staining in the images was quantified using HistoFAXS (TissuGnostics). Data in the graph are means ± SD of the intensity measured (n = 5 per group, P = 0.3477 (non-polyp) and 0.3559 (polyp)). Scale bar, 50 µm.

b. Number of proliferating cells in the polyps in the small intestine. 5-bromo-2'-deoxyuridine (BrdU) incorporation was performed for 2 hr after intraperitoneal injection (0.1 ml injection of 10 mM BrdU in PBS per 10g body weight). Paraffin tissue sections were immunostained with anti-BrdU antibody. Cell numbers were counted using the Image J program. Data in the graph are means ± SEM of the number of BrdU-positive cells per polyp (n = 4 mice for *PrxII*$^{+/+}$, n = 3 mice for *PrxII*$^{-/-}$, P = 0.4816 with two-tailed t-test). Scale bar, 50 µm.

c. Migration of epithelial cells in the villi of small intestine and colon. BrdU incorporation was performed for 30 hr after intraperitoneal injection. Paraffin tissue sections were immunostained with anti-BrdU antibody. Cell migration was determined by measuring the length of the most migrating cell from bottom of crypt using NIS-Elements BR3.2 imaging software (Nikon). Data in the graph are means ± SEM of the percent of the length of most migrating cell per total length of villus (V) plus crypt (C) or crypt (n = 3 mice for *PrxII*$^{+/+}$, n = 5 mice for *PrxII*$^{-/-}$, P = 0.6159 (small intestine) and P = 0.1851 (colon) with two-tailed t-test). A representative image is shown. N.S., not significant. Scale bar, 50 µm.

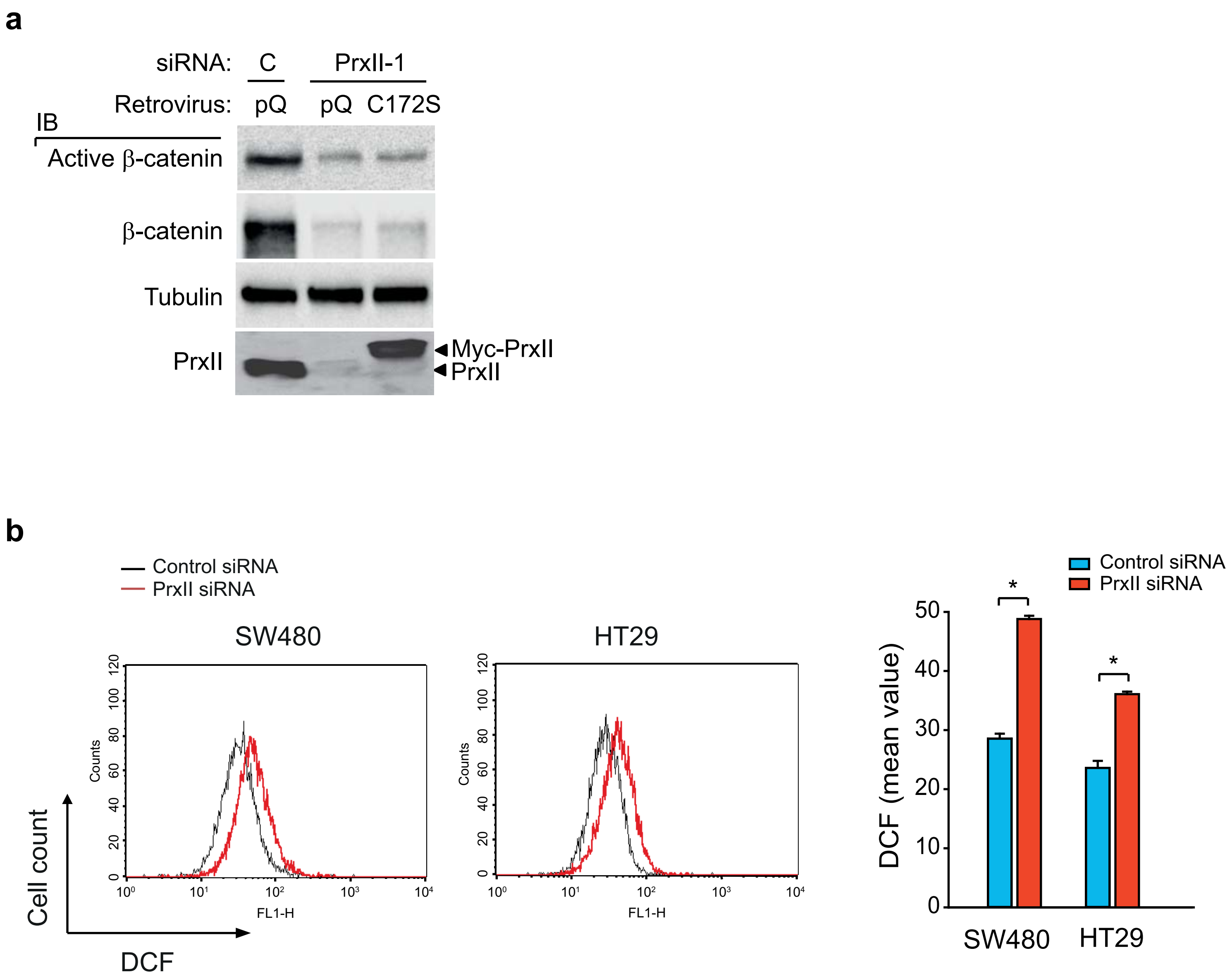

Supplementary Fig. 3. APC-dependent cellular H_2O_2 levels in CRC cells

a. HT29 cells were transfected with control or PrxII-1 siRNa for 24 hr and then infected with retroviruses expressing siRNA-resistant PrxII-C172S mutant with a Myc tag for additional 24 hr. Empty retrovirus (pQ) was used for control. The cell lysate were subjected to Western blotting for indicated proteins.

b. Intracellular H_2O_2 level was increased by PrxII depletion in SW480 and HT29 cells. CRC cells were transfected with control and PrxII-siRNA for 24 hr. Next day, cells were rinsed with a phenol-red-free basal medium and incubated with 5 μM 5,6-chloromethyl-2',7'-dichlorohydrofluorescein diacetate at 37 °C for 30 min. The cells were trypsinized and analyzed with a flow cytometry. Data in the graph are means ± SD of dichlorofluorescein (DCF) fluorescence values from three independent experiments ($^{*}P < 0.001$)

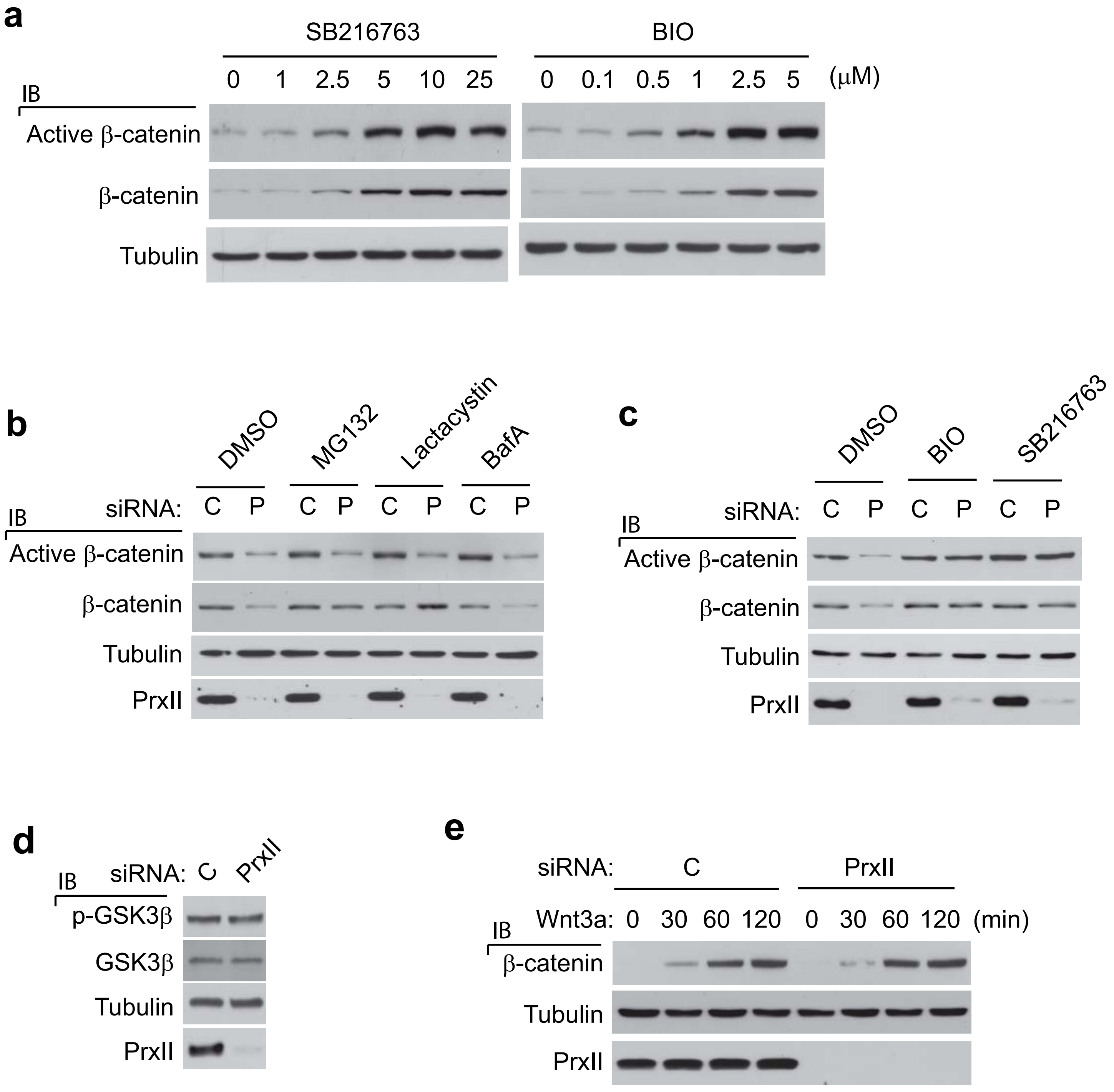

a
SB216763
BIO
0 1 2.5 5 10 25
0 0.1 0.5 1 2.5 5 (μM)
IB
Active β-catenin
β-catenin
Tubulin
b
DMSO
MG132
Lactacystin
BafA
siRNA: C P C P C P C P
IB
Active β-catenin
β-catenin
Tubulin
PrxII
c
DMSO
BIO
SB216763
siRNA: C P C P C P
IB
Active β-catenin
β-catenin
Tubulin
PrxII
d
IB
siRNA: C PrxII
p-GSK3β
GSK3β
Tubulin
PrxII
e
siRNA: C PrxII
Wnt3a: 0 30 60 120 0 30 60 120 (min)
IB
β-catenin
Tubulin
PrxII

Supplementary Fig. 4. Effect of proteasomal and GSK3β inhibition on PrxII-dependent β-catenin degradation in APC-mutant CRC cells.

a. Titration of GSK3β inhibitors (SB216763 and BIO) for β-catenin stabilization. HEK293 cells were treated with increasing concentrations of the indicated compounds and subjected to immunoblot (IB) analysis for total and active β-catenins. The optimal concentrations for maximum inhibition were determined to be 10 μM and 2.5 μM for SB216763 and BIO, respectively. Active β-catenin represents the transcriptionally active form unphosphorylated on Ser37/Thr41.

b and **c**. HT29 cells were transfected with control (C) and PrxII-1 siRNA for 24 hr and then treated with proteasome and GSK3β inhibitors (MG132, 25μM; lactacystin, 1μM; bafilomycin A1 (BafA), 100 nM; SB216763, 10 μM; BIO, 2.5 μM) for further 24 hr. The lysosomal inhibitor BafA did not restore the β-catenin level. Control vehicle was dimethyl sulfoxide (DMSO).

d. SW480 cells were transfected with control and PrxII-1 siRNA for 24 hr and immunoblotted for Y216 phosphorylation, an indicator of GSK3β activation.

e. HEK293 cells were transfected with control and PrxII-1 siRNA and then treated with Wnt3a (100 ng/ml) for the indicated times. The cytosolic fraction was isolated by ultracentrifugation and subjected to immunoblot analysis. A representative blot from three independent experiments is shown.

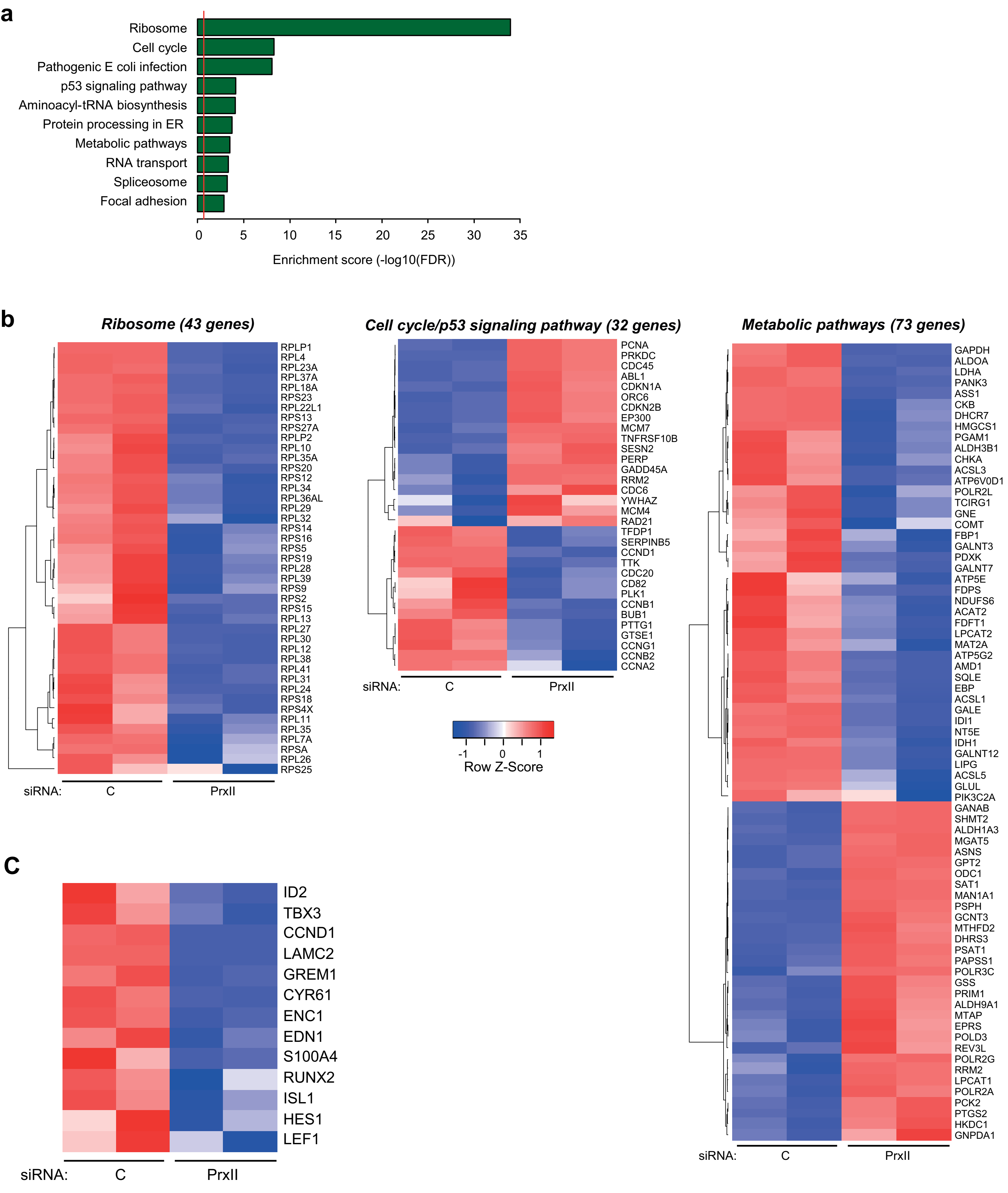
a
Ribosome
Cell cycle
Pathogenic E coli infection
p53 signaling pathway
Aminoacyl-tRNA biosynthesis
Protein processing in ER
Metabolic pathways
RNA transport
Spliceosome
Focal adhesion
0 5 10 15 20 25 30 35
Enrichment score (-log10(FDR))
b
Ribosome (43 genes)
RPLP1 RPL4 RPL23A RPL37A RPL18A RPS23 RPL22L1 RPS13 RPS27A RPLP2 RPL10 RPL35A RPS20 RPS12 RPL34 RPL36AL RPL29 RPL32 RPS14 RPS16 RPS5 RPS19 RPL28 RPL39 RPS9 RPS2 RPS15 RPL13 RPL27 RPL30 RPL12 RPL38 RPL41 RPL31 RPL24 RPS18 RPS4X RPL11 RPL35 RPL7A RPSA RPL26 RPS25
siRNA: C PrxII
Cell cycle/p53 signaling pathway (32 genes)
PCNA PRKDC CDC45 ABL1 CDKN1A ORC6 CDKN2B EP300 MCM7 TNFRSF10B SESN2 PERP GADD45A RRM2 CDC6 YWHAZ MCM4 RAD21 TFDP1 SERPINB5 CCND1 TTK CDC20 CD82 PLK1 BUB1 CCNB1 PTTG1 GTSE1 CCNG1 CCNB2 CCNA2
siRNA: C PrxII
-1 0 1
Row Z-Score
Metabolic pathways (73 genes)
GAPDH ALDOA LDHA PANK3 ASS1 CKB DHCR7 HMGCS1 PGAM1 ALDH3B1 CHKA ACSL3 ATP6V0D1 POLR2L TCIRG1 GNE COMT FBP1 GALNT3 PDXK GALNT7 ATP5E FDPS NDUFS6 ACAT2 FDFT1 LPCAT2 MAT2A ATP5G2 AMD1 SQLE EBP ACSL1 GALE IDI1 NT5E IDH1 GALNT12 LIPG ACSL5 GLUL PIK3C2A GANAB SHMT2 ALDH1A3 MGAT5 ASNS GPT2 ODC1 SAT1 MAN1A1 PSPH GCNT3 MTHFD2 DHRS3 PSAT1 PAPSS1 POLR3C GSS PRIM1 ALDH9A1 MTAP EPRS POLD3 REV3L POLR2G RRM2 LPCAT1 POLR2A PCK2 PTGS2 HKDC1 GNPDA1
siRNA: C PrxII
c
ID2 TBX3 CCND1 LAMC2 GREM1 CYR61 ENC1 EDN1 S100A4 RUNX2 ISL1 HES1 LEF1
siRNA: C PrxII

Supplementary Fig. 5. Analysis of differentially expressed genes in HT29 cells after PrxII depletion.

RNA-Seq was performed as described in *Methods*. The 789 differentially-expressed genes (DEG) were identified with false discovery rate (FDR) cut-off 0.05 and then analyzed for KEGG pathway.

a. Bar plot showing top 10 KEGG pathways with significant FDR values.

b. Heat map depicting the list of DEGs belonging to representative KEGG pathways. Duplicate experimental data sets are shown in parallel.

c. Heat map depicting the significant downregulation of β-catenin/TCF-dependent gene expression by PrxII depletion in SW480 cells. Duplicate experiments are shown in parallel.

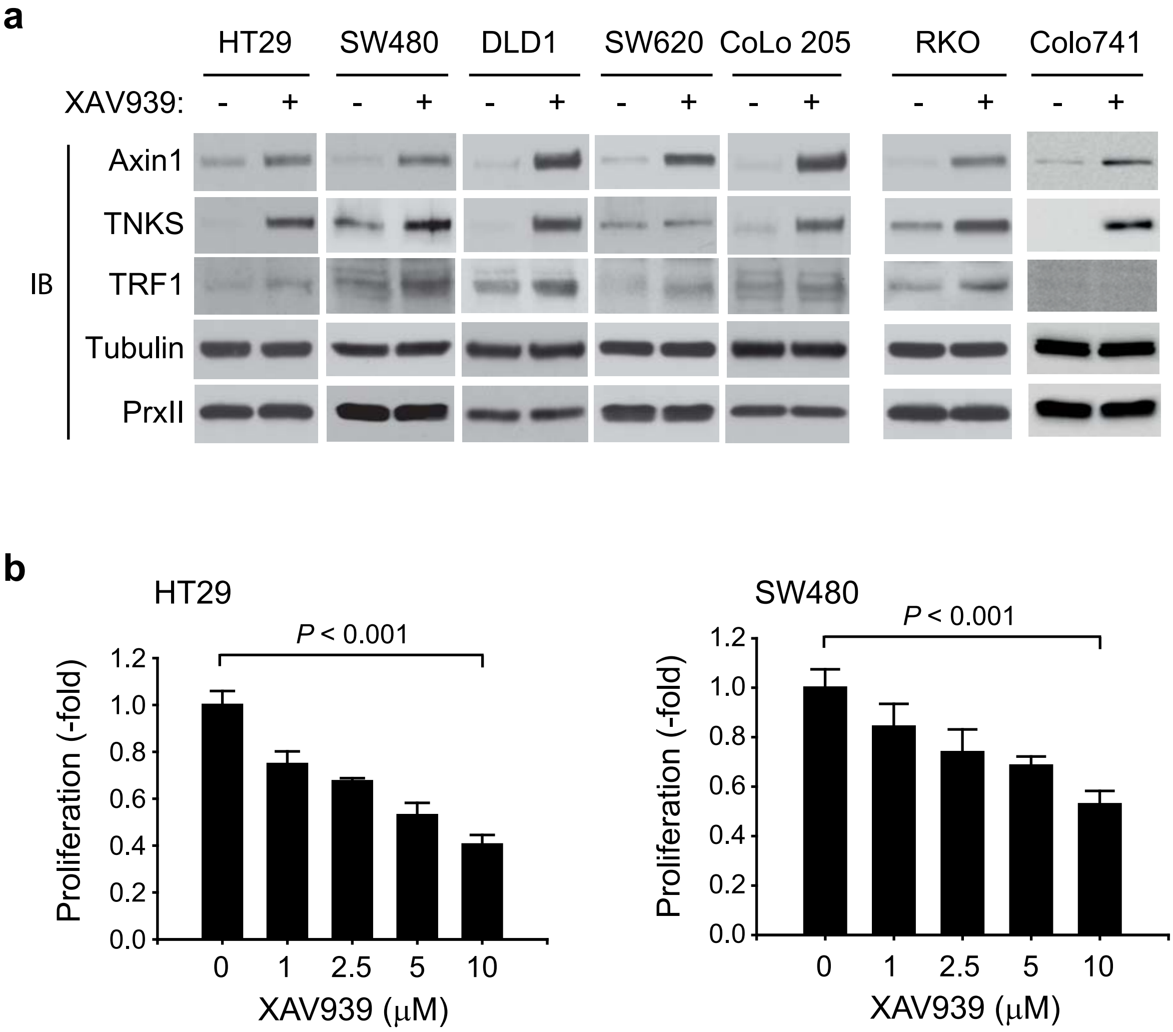

Supplementary Fig 6. Levels of TNKS and its substrate proteins in APC-mutant CRC cells treated with a TNKS inhibitor XAV939.

a. CRC cells were treated with DMSO vehicle control or TNKS inhibitor XAV939 (1μM) for 18 hr and then subjected to Western blot analysis for indicted proteins. HT29 and CoLo205 cells were exceptionally treated with 5 μM XAV939 for 18 hr. Immunoblot (IB) is a representative of two independent experiments with the same results.

b. HT29 and SW480 cells were treated with indicated concentrations of XAV939 for 36 hr. Cell number were measured with WST-1 reagent (Roche) according to manufacturer's protocol. Data in the graph show means ± SD of fold changes versus untreated sample (n = 3). P values are indicated.

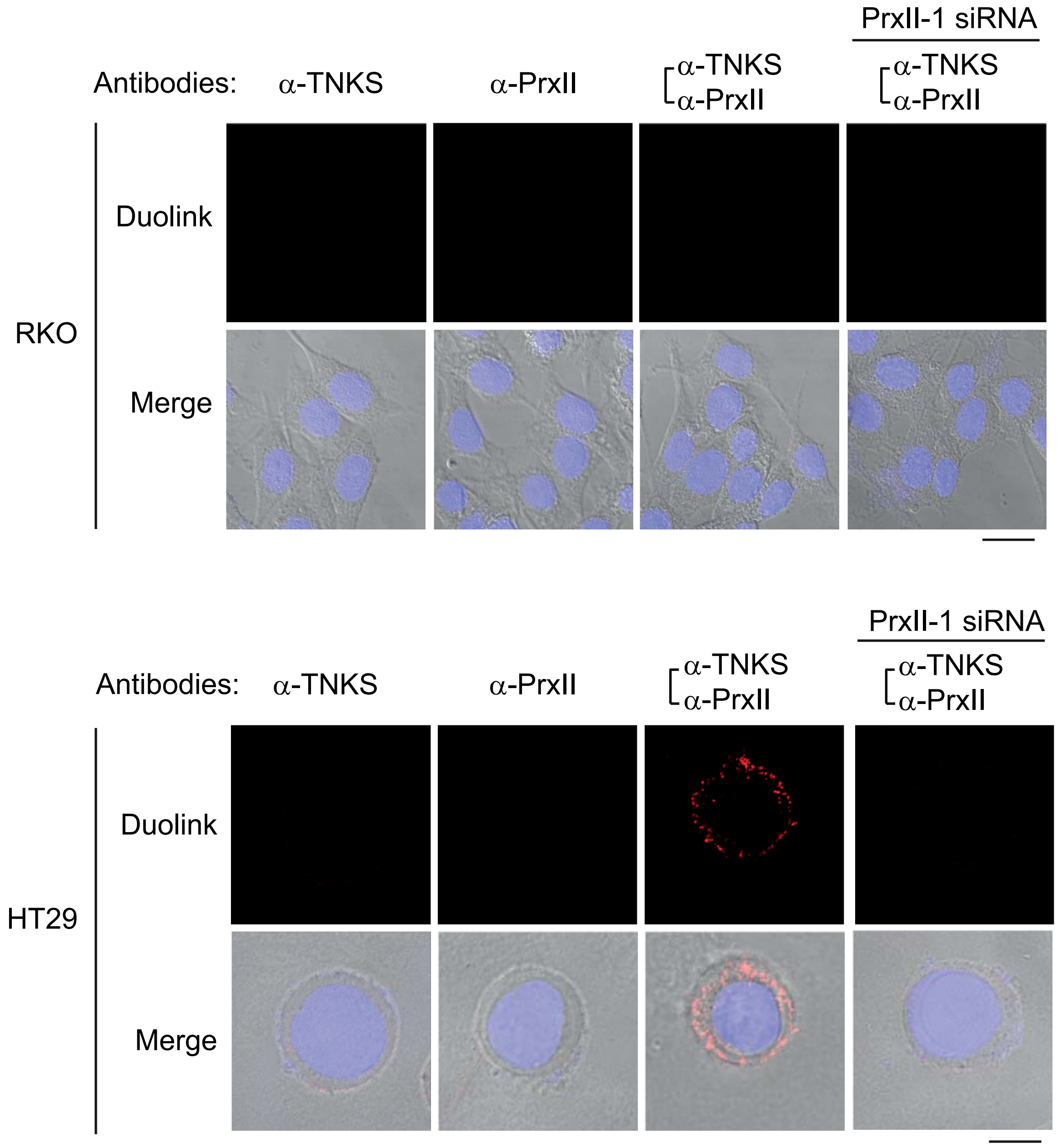

Supplementary Fig. 7. Proximal ligation assay shows direct interaction between TNKS and PrxII in the cytosol of APC-mutant CRC cells.

An *in situ* proximal ligation assay to detect binding between TNKS and PrxII proteins was performed in RKO and HT29 cells using DuoLink® *in situ* probes with the indicated antibodies. DuoLink Red fluorescence was detected only by the combination of anti-TNKS and anti-PrxII antibodies, not by single antibodies, which suggests a specific interaction between TNKS and PrxII. As a negative control, CRC cells were transfected with PrxII-1 siRNA. DIC and DAPI-stained images were merged with DuoLink fluorescence images (Merge). A representative image from three independent experiments is shown. Scale bar, 20 μm (RKO) and 10 μm (HT29).

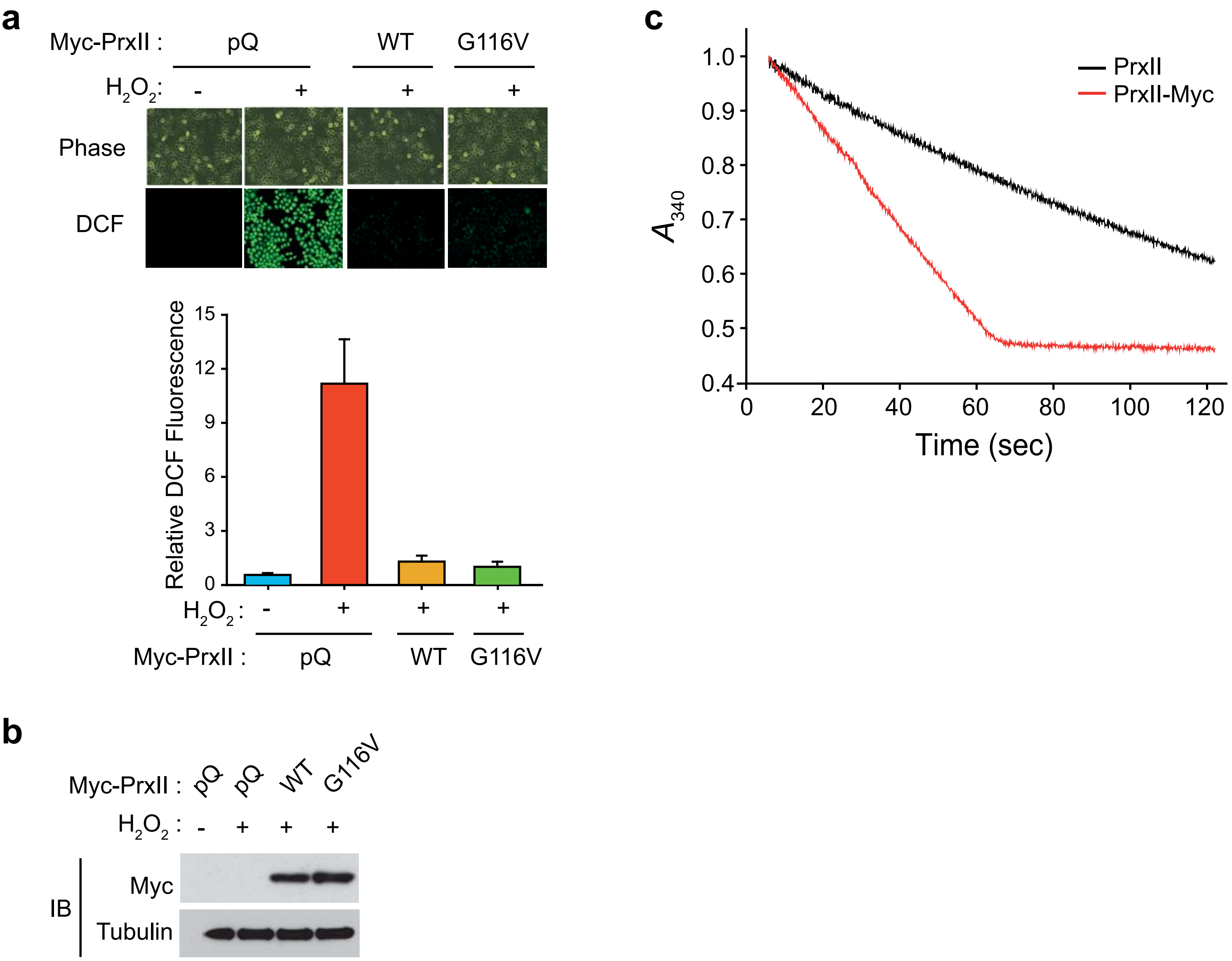

Supplementary Fig. 8. Peroxidase activity of the PrxII G116V mutant and PrxII with a Myc tag.

a. Cellular peroxidase activities were measured using an H_2O_2-sensitive fluorescence probe, 5,6-chloromethyl-2′,7′-dichlorodihydrofluorescein diacetate. HEK293 cells were infected with retroviruses expressing WT and G116V mutant of Myc-tagged PrxII for 24 hr. The cells were treated with 100μM H_2O_2 for 10 min, washed with a phenol-red-free basal medium, and incubated with 5 μM 5,6-chloromethyl-2′,7′-dichlorodihydrofluorescein diacetate at 37°C for 5 min. The dichlorofluorescein (DCF) fluorescence images were taken by a fluorescence microscope (Axiovert 200 Basic standard, Zeiss, Germany). The relative DCF fluorescence was calculated by averaging the fluorescence levels from 50 - 80 cells after subtracting the background fluorescence. Data in the graph are means ± SD of relative DCF fluorescence values from three independent experiments.

b. Immunoblotting (IB) was performed for checking the expression of indicated proteins. The empty retrovirus (pQ) was used for control.

c. Peroxidase activity assay using purified recombinant proteins of PrxII and PrxII with a Myc tag (PrxII-Myc) was performed in a reaction mixture containing 50mM HEPES (pH7.0), 4.3 μM human Trx, 0.165 μM rat liver TrxR, 200 μM NADPH, 100μM H_2O_2, and 3.4μM PrxII.

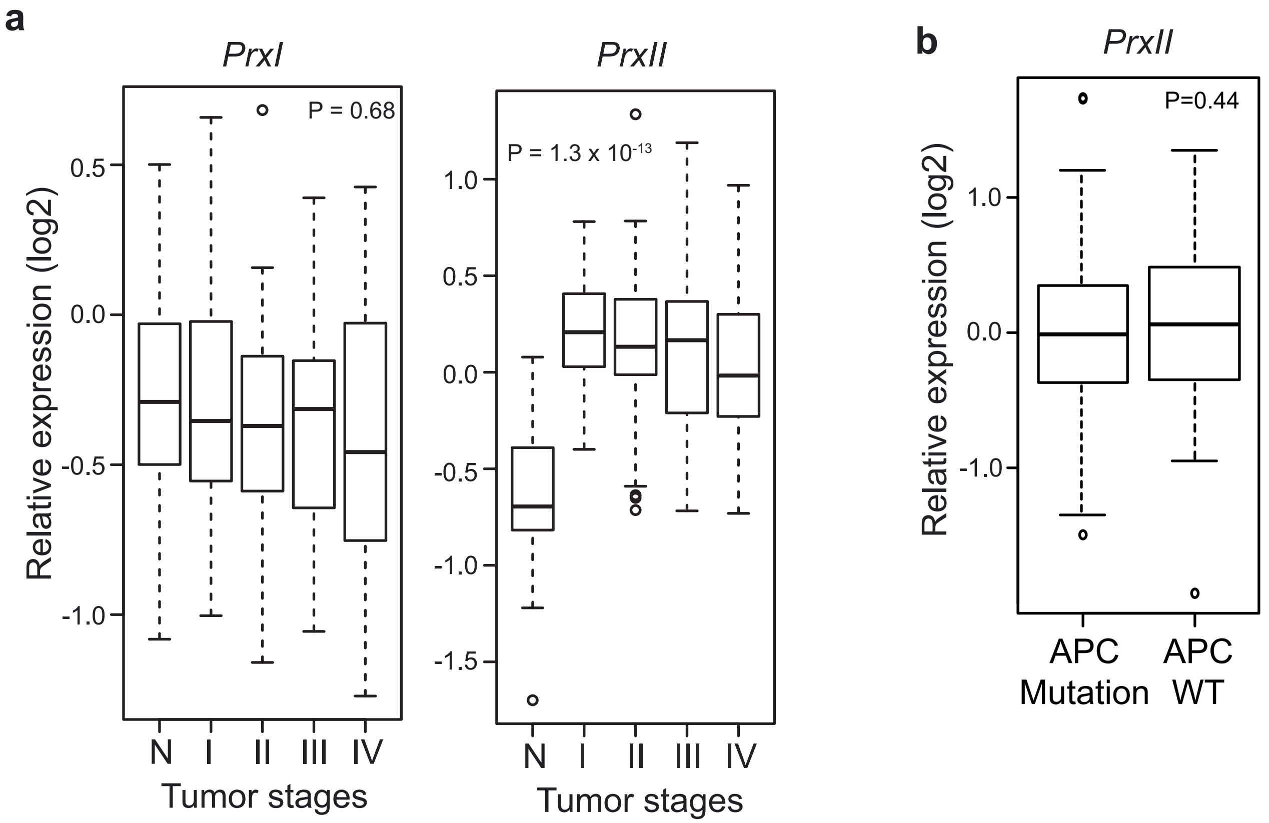

Supplementary Fig. 9. Increased expression of *PrxII* genes in patients with colorectal cancer.

a. Tumor stage-dependent expression of *PrxI* and *PrxII* genes, N, normal.

b. Expression levels of PrxII gene in colon cancer patients with APC WT (*n* = 45) and APC mutation (*n* = 104). The GA platform data (*n* = 149) from TCGA COAD database were used for analysis.

Box plot represents the average expression of PrxI and PrxII ± SD. The horizontal line indicates the median value.

Supplementary Fig 10 Uncropped images of all the western blot data

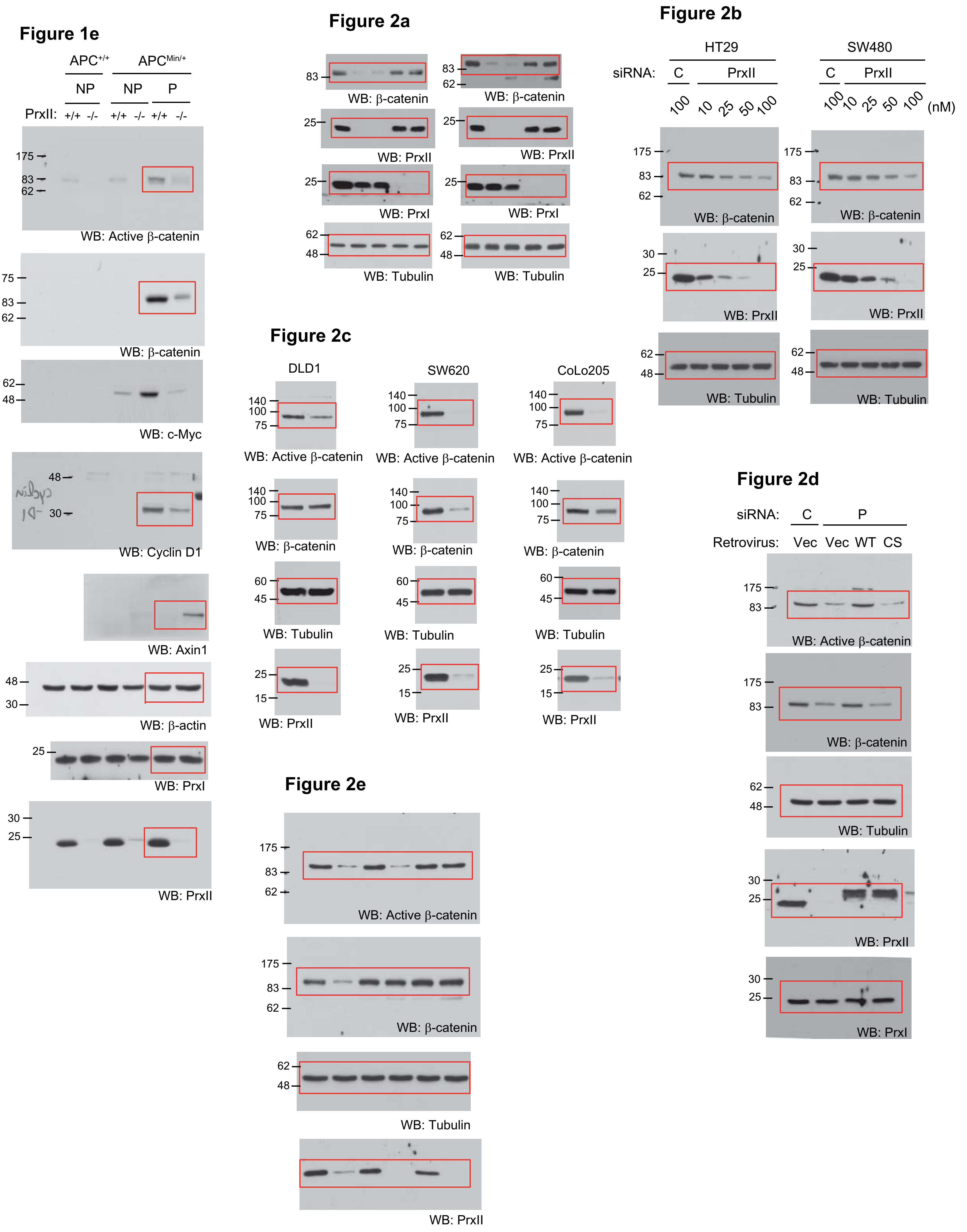

Supplementary Fig 10 Continued

Figure 3a

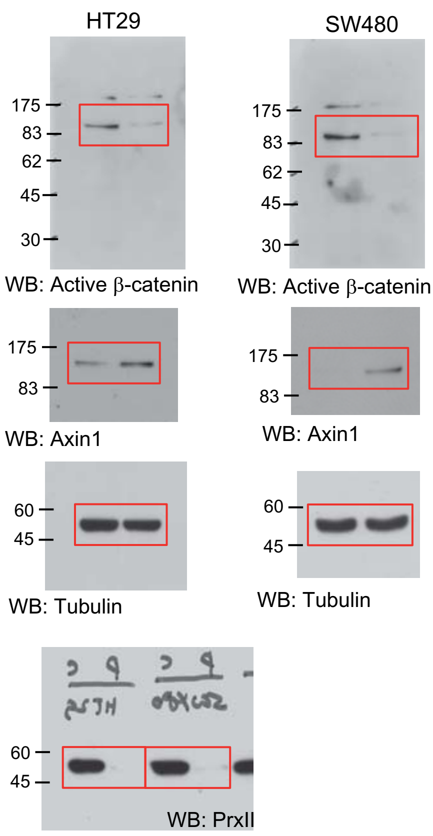

Figure 3b

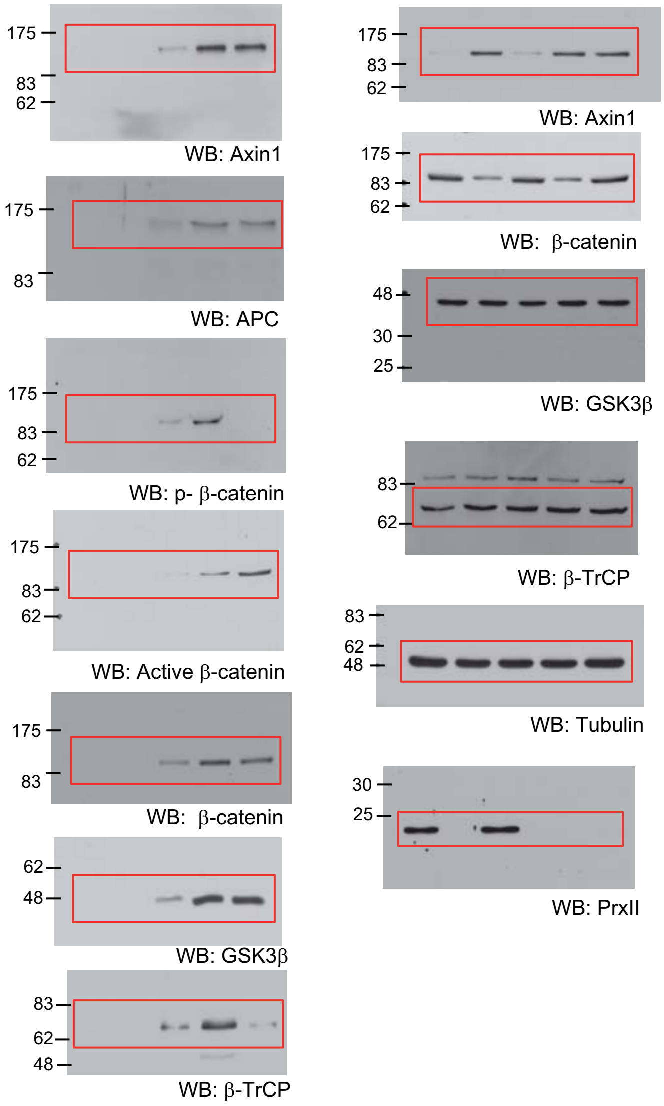

Figure 3c

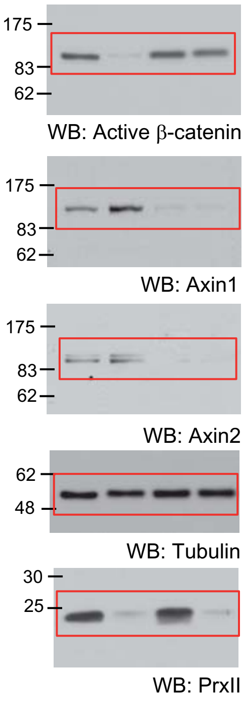

Figure 3d

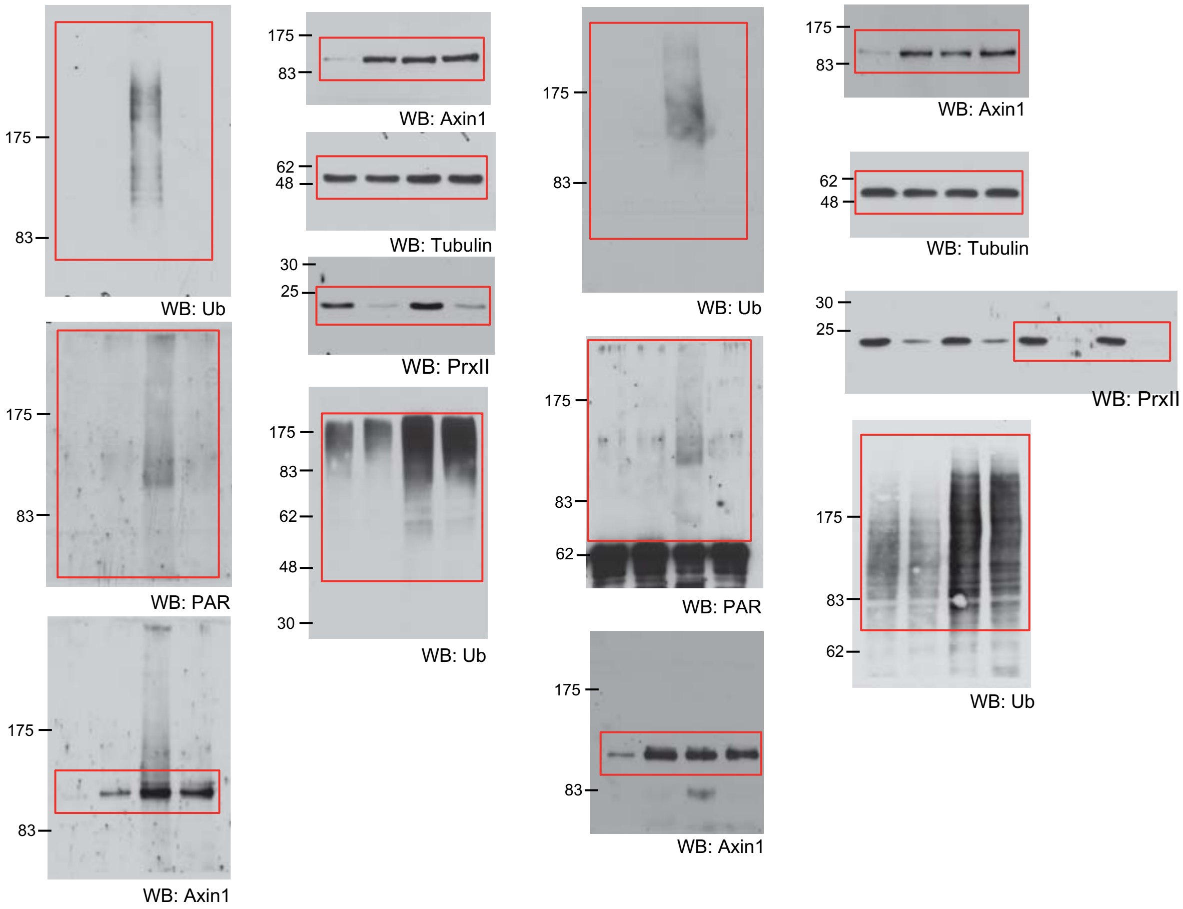

Supplementary Fig 10 Continued

Figure 4a/b

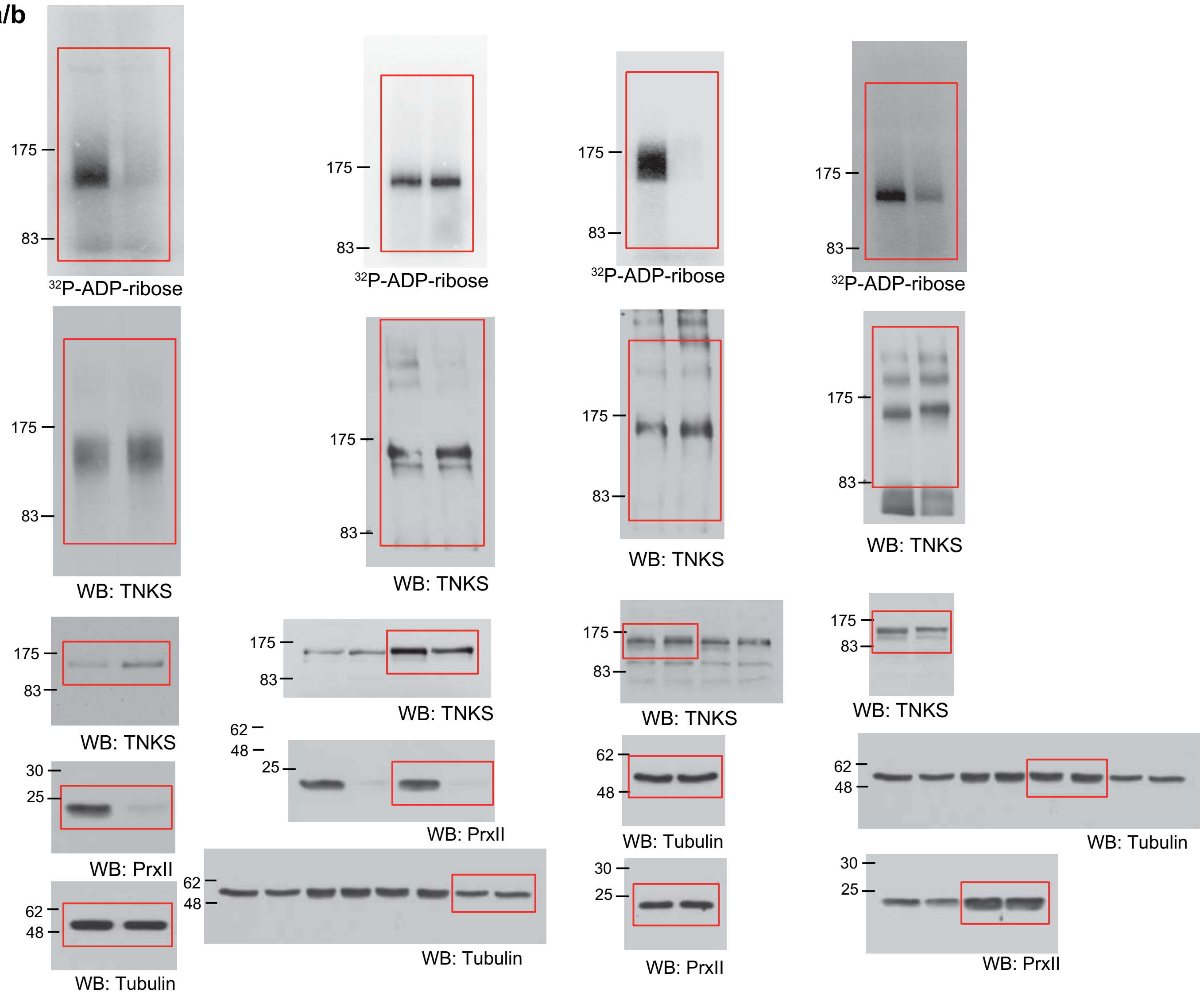

Figure 4C

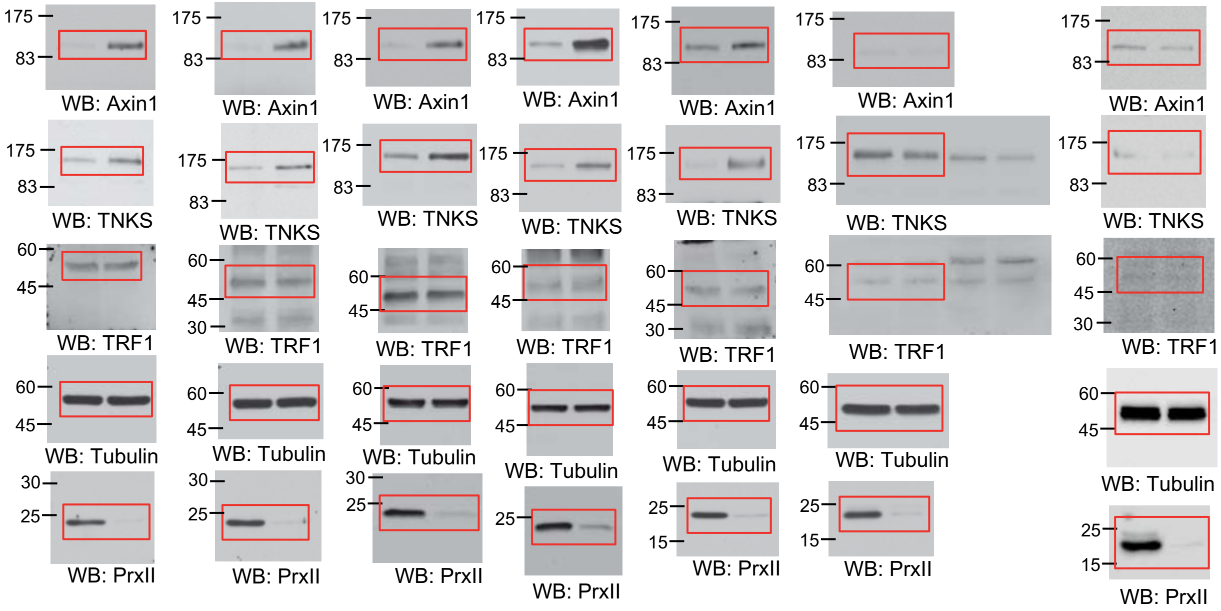

Figure 4d

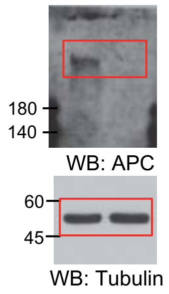

Supplementary Fig 10 Continued

Figure 4e

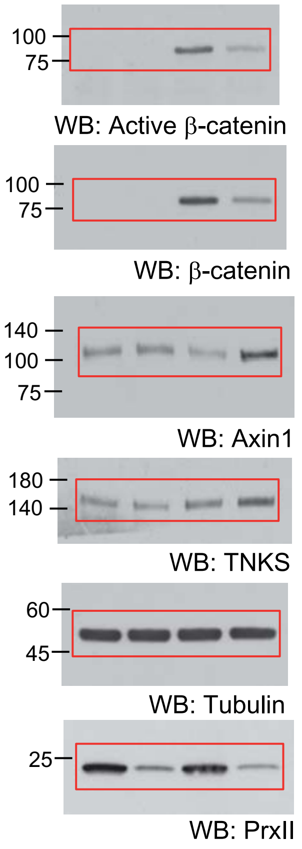

Figure 4f

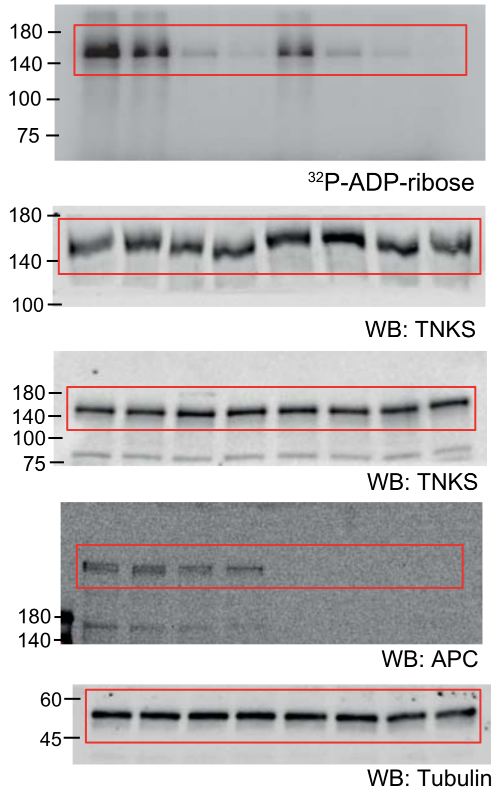

Figure 4g

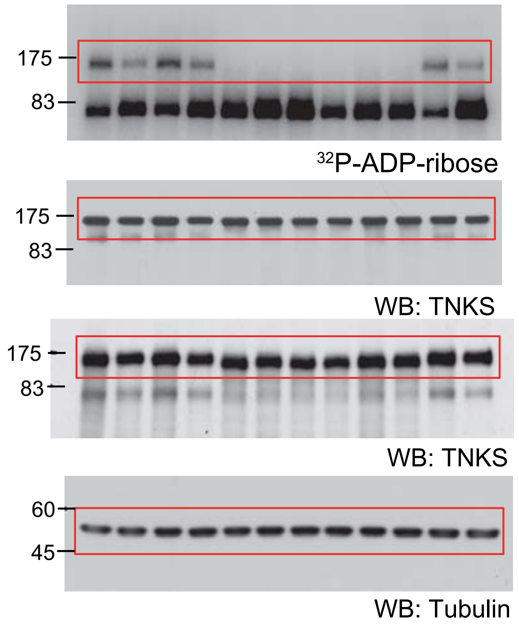

Figure 4h

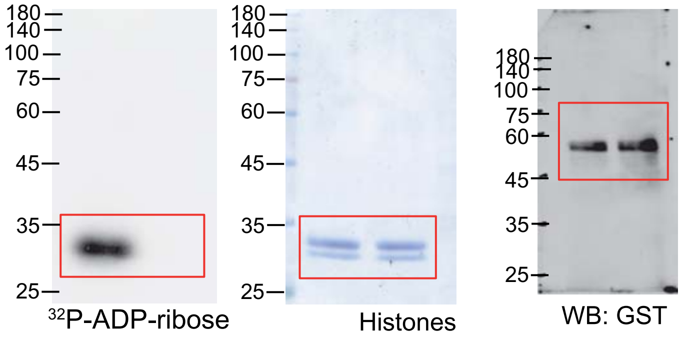

Supplementary Fig 10 Continued

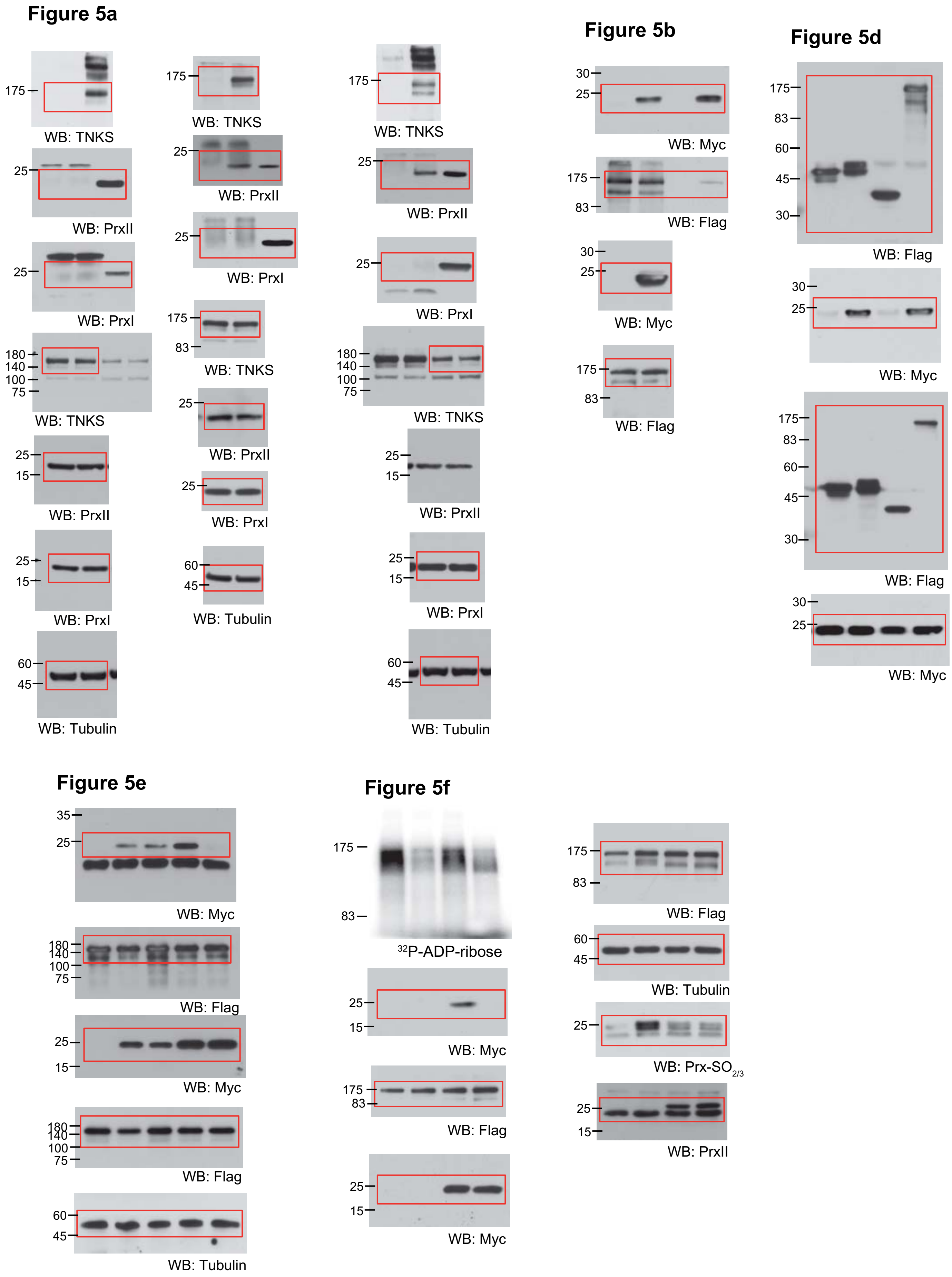

Supplementary Fig 10 Continued

Supplementary Figure 3a

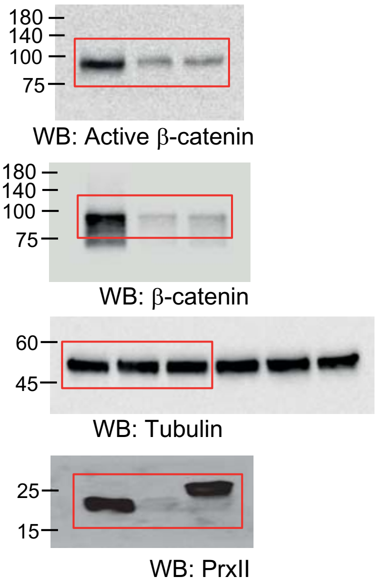

Supplementary Figure 4a

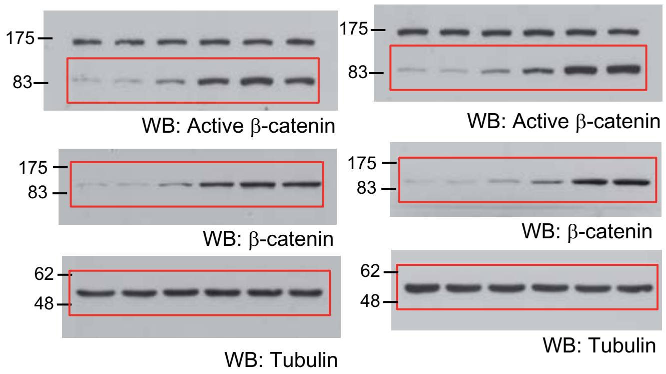

Supplementary Figure 4b

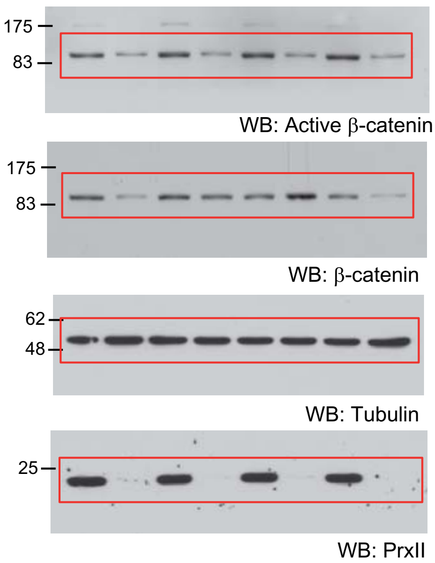

Supplementary Figure 4c

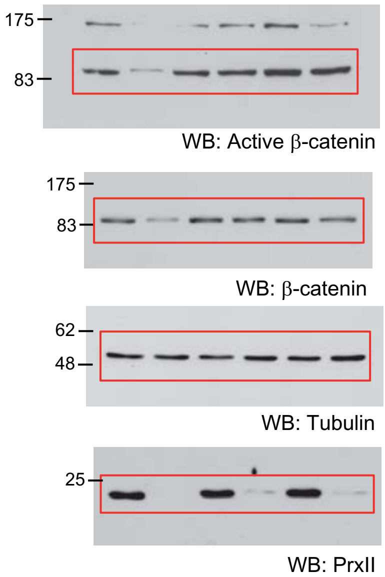

Supplementary Figure 4e

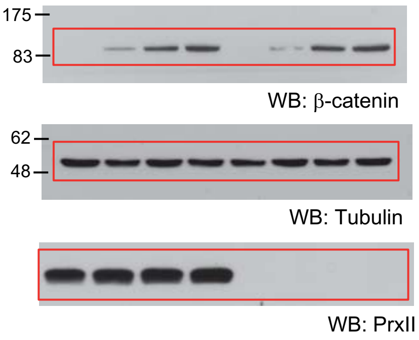

Supplementary Figure 4d

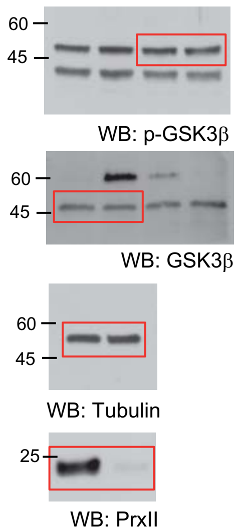

Supplementary Figure 6

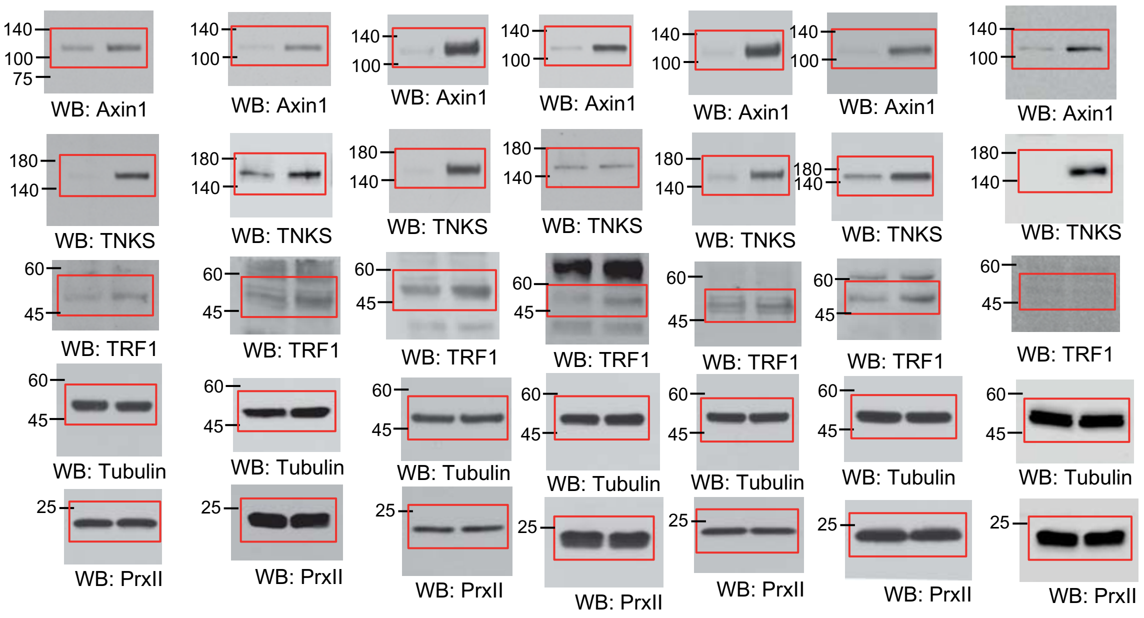

Supplement: Supplementary file 1 — Supplementary Information [file 41467_2017_54_MOESM1_ESM.pdf]
